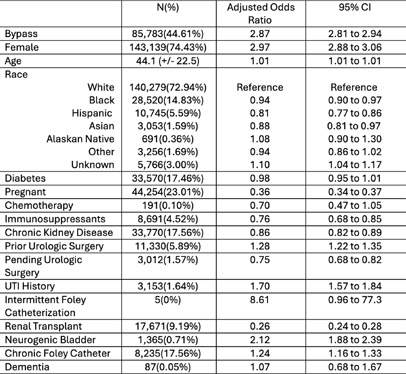# Implementation of Urinalysis with Reflex Culture Order Sets Associated with Fewer Outpatient Antibiotics for Urinary Tract Infections

**DOI:** 10.1017/ash.2025.274

**Published:** 2025-09-24

**Authors:** Lauren Johansen, Charles Oertil, Tom Talbot, Sophie Katz, Jennifer Cihlar, Rebecca Stern, Nico Herrera, Titus Daniels, Kaitlyn Reasoner, Andrea Ito, Michael Zou, Sharon Onguti, Milner Staub

**Affiliations:** 1 Vanderbilt University; 2 Vanderbilt University School of Medicine; 3 Vanderbilt University Medical Center; 4 Vanderbilt University Medical Center; 5 Vanderbilt University Medical Center; 6 VUMC; 7 Vanderbilt University Medical Center; 8 Vanderbilt University Medical Center; 9 Vanderbilt University; 10 Vanderbilt University; 11 Vanderbilt University Medical Center

## Abstract

**Background:** Urinalysis with reflex culture order sets (reflex order set) require urinalyses to meet specific criteria before triggering a culture to reduce unnecessary urine cultures and inappropriate treatment of asymptomatic bacteriuria (ASB). A reflex order set was designed and implemented at a large academic medical center in 2016 and updated in June 2022 to require clinicians to select which pre-specified exemption the patient met to bypass the reflex order set and order a urine culture. We aimed to assess the association between reflex order set bypass and antibiotic prescribing for urinary tract infections (UTIs) in outpatient encounters. **Methods:** Patient demographics, co-morbidities, encounter diagnoses, and treatment data, including required antibiotic indications, were extracted from all outpatient healthcare system adult and pediatric patient encounters utilizing the reflex order set. Using multivariable logistic aggression, we assessed associated odds with 95% confidence intervals (95% CI) of bypassing the reflex order set and antibiotic prescribing for UTI. **Results:** From June 2022 to June 2024, 192,310 encounters met inclusion criteria. After adjusting for patient factors, bypassing the reflex order set was associated with higher odds (2.87 95% CI: 2.81 to 2.94) of antibiotic prescribing for UTI. Increasing age, female gender, indwelling catheter, history of urological surgery, UTI, and neurogenic bladder were associated with increased prescribing. Being on immunosuppression, pregnancy, pending urological surgery, renal transplant status and chronic kidney disease were associated with reduced odds of antibiotic prescribing (Table 1). **Discussion:** Urinalysis reflex order set implementation in a large ambulatory clinic system was associated with lower likelihood of antibiotic prescribing for UTI. Further analysis will evaluate accuracy of selected bypass indications and appropriateness of antibiotic prescriptions to identify opportunities for optimizing this intervention.

**Figure:**